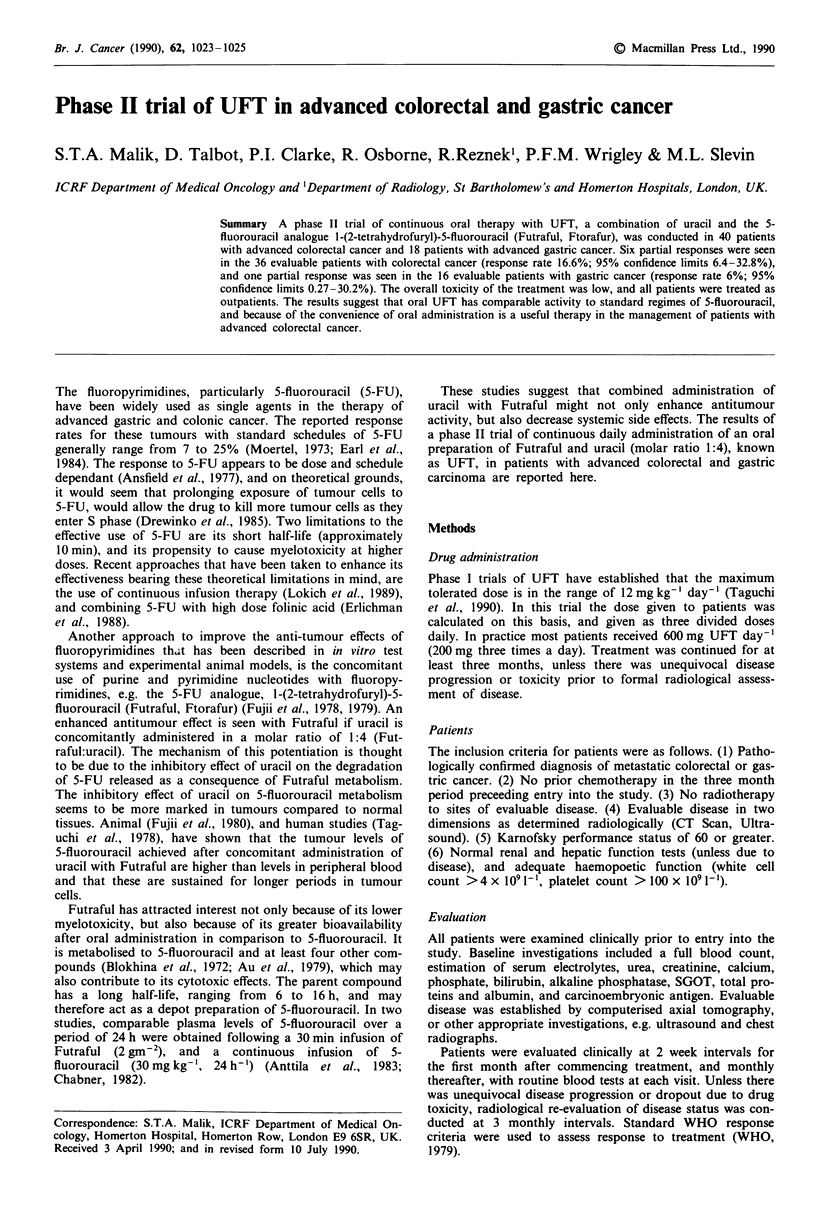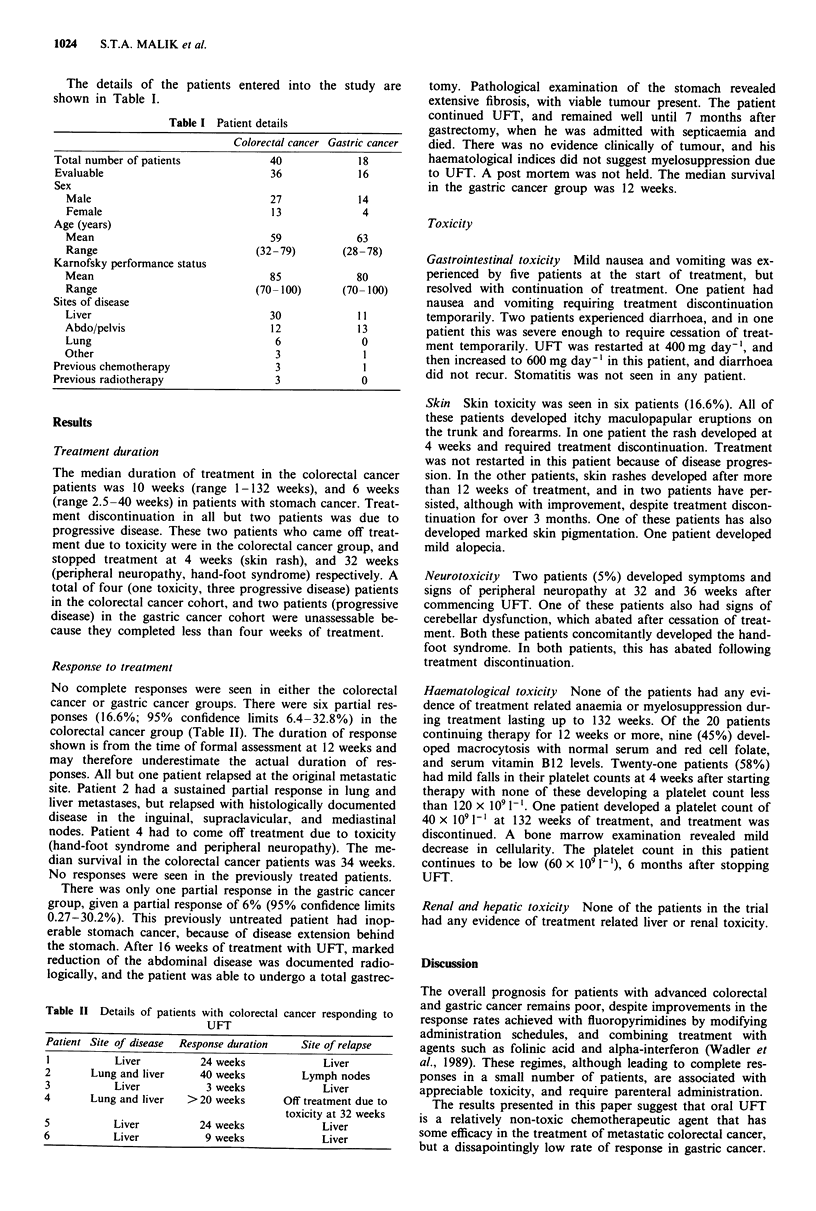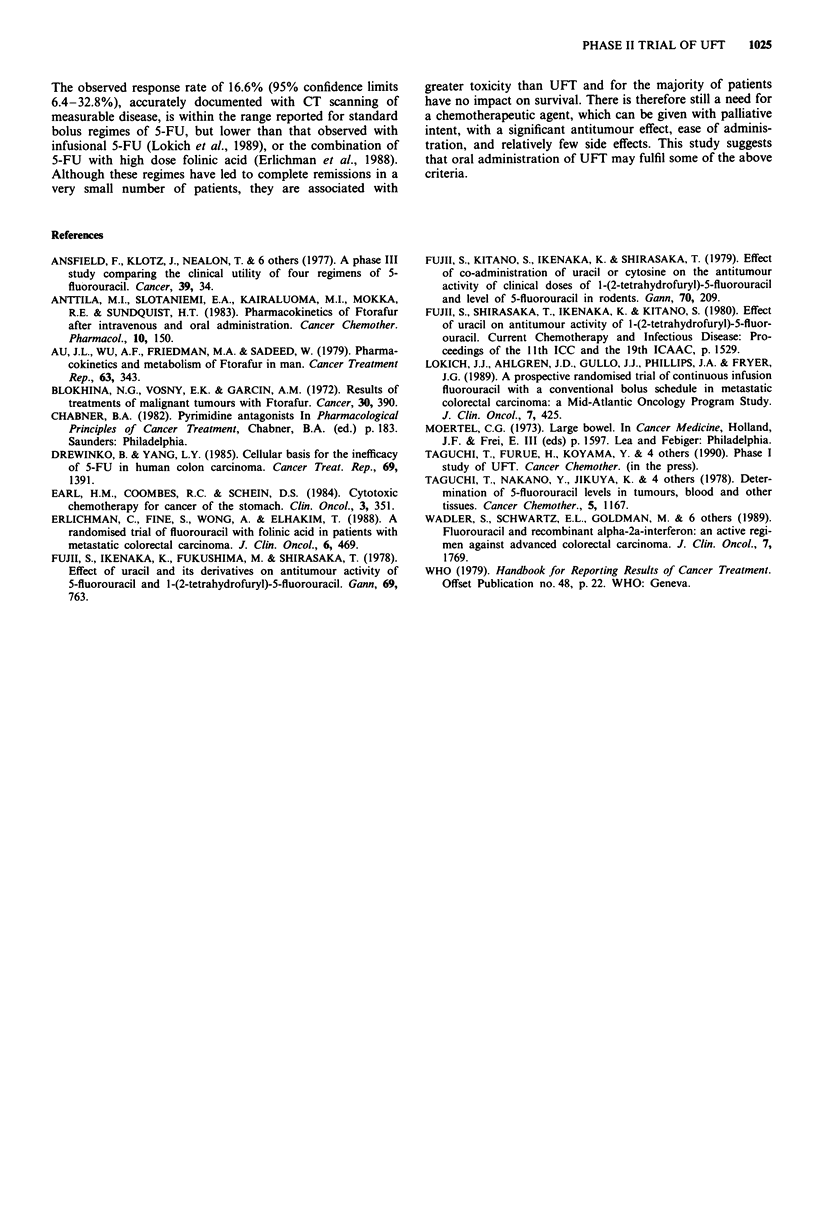# Phase II trial of UFT in advanced colorectal and gastric cancer.

**DOI:** 10.1038/bjc.1990.431

**Published:** 1990-12

**Authors:** S. T. Malik, D. Talbot, P. I. Clarke, R. Osborne, R. Reznek, P. F. Wrigley, M. L. Slevin

**Affiliations:** ICRF Department of Medical Oncology, St. Bartholomew's, Hospital, London, UK.

## Abstract

A phase II trial of continuous oral therapy with UFT, a combination of uracil and the 5-fluorouracil analogue 1-(2-tetrahydrofuryl)-5-fluorouracil (Futraful, Ftorafur), was conducted in 40 patients with advanced colorectal cancer and 18 patients with advanced gastric cancer. Six partial responses were seen in the 36 evaluable patients with colorectal cancer (response rate 16.6%; 95% confidence limits 6.4-32.8%), and one partial response was seen in the 16 evaluable patients with gastric cancer (response rate 6%; 95% confidence limits 0.27-30.2%). The overall toxicity of the treatment was low, and all patients were treated as outpatients. The results suggest that oral UFT has comparable activity to standard regimes of 5-fluorouracil, and because of the convenience of oral administration is a useful therapy in the management of patients with advanced colorectal cancer.


Br. J. Cancer (1990), 62, 1023-1025 © Macmillan Press Ltd., 1990

### Phase II trial of UFT in advanced colorectal and gastric cancer

S.T.A. Malik, D. Talbot, P.I. Clarke, R. Osborne, R.Reznek[1], P.F.M. Wrigley & M.L. Slevin

*ICRF Department of Medical Oncology and [1]Department of Radiology, St Bartholomew's and Homerton Hospitals, London, UK.*

**Summary** A phase II trial of continuous oral therapy with UFT, a combination of uracil and the 5-fluorouracil analogue 1-(2-tetrahydrofuryl)-5-fluorouracil (Futraful, Ftorafur), was conducted in 40 patients with advanced colorectal cancer and 18 patients with advanced gastric cancer. Six partial responses were seen in the 36 evaluable patients with colorectal cancer (response rate 16.6%; 95% confidence limits 6.4-32.8%), and one partial response was seen in the 16 evaluable patients with gastric cancer (response rate 6%; 95% confidence limits 0.27-30.2%). The overall toxicity of the treatment was low, and all patients were treated as outpatients. The results suggest that oral UFT has comparable activity to standard regimes of 5-fluorouracil, and because of the convenience of oral administration is a useful therapy in the management of patients with advanced colorectal cancer.

The fluoropyrimidines, particularly 5-fluorouracil (5-FU), have been widely used as single agents in the therapy of advanced gastric and colonic cancer. The reported response rates for these tumours with standard schedules of 5-FU generally range from 7 to 25% (Moertel, 1973; Earl *et al.*, 1984). The response to 5-FU appears to be dose and schedule dependant (Ansfield *et al.*, 1977), and on theoretical grounds, it would seem that prolonging exposure of tumour cells to 5-FU, would allow the drug to kill more tumour cells as they enter S phase (Drewinko *et al.*, 1985). Two limitations to the effective use of 5-FU are its short half-life (approximately 10 min), and its propensity to cause myelotoxicity at higher doses. Recent approaches that have been taken to enhance its effectiveness bearing these theoretical limitations in mind, are the use of continuous infusion therapy (Lokich *et al.*, 1989), and combining 5-FU with high dose folinic acid (Erlichman *et al.*, 1988).

Another approach to improve the anti-tumour effects of fluoropyrimidines that has been described in *in vitro* test systems and experimental animal models, is the concomitant use of purine and pyrimidine nucleotides with fluoropyrimidines, e.g. the 5-FU analogue, 1-(2-tetrahydrofuryl)-5-fluorouracil (Futraful, Ftorafur) (Fujii *et al.*, 1978, 1979). An enhanced antitumour effect is seen with Futraful if uracil is concomitantly administered in a molar ratio of 1:4 (Futraful:uracil). The mechanism of this potentiation is thought to be due to the inhibitory effect of uracil on the degradation of 5-FU released as a consequence of Futraful metabolism. The inhibitory effect of uracil on 5-fluorouracil metabolism seems to be more marked in tumours compared to normal tissues. Animal (Fujii *et al.*, 1980), and human studies (Taguchi *et al.*, 1978), have shown that the tumour levels of 5-fluorouracil achieved after concomitant administration of uracil with Futraful are higher than levels in peripheral blood and that these are sustained for longer periods in tumour cells.

Futraful has attracted interest not only because of its lower myelotoxicity, but also because of its greater bioavailability after oral administration in comparison to 5-fluorouracil. It is metabolised to 5-fluorouracil and at least four other compounds (Blokhina *et al.*, 1972; Au *et al.*, 1979), which may also contribute to its cytotoxic effects. The parent compound has a long half-life, ranging from 6 to 16 h, and may therefore act as a depot preparation of 5-fluorouracil. In two studies, comparable plasma levels of 5-fluorouracil over a period of 24 h were obtained following a 30 min infusion of Futraful (2 $gm^{-2}$), and a continuous infusion of 5-fluorouracil (30 mg $kg^{-1}$, 24 $h^{-1}$) (Anttila *et al.*, 1983; Chabner, 1982).

Correspondence: S.T.A. Malik, ICRF Department of Medical Oncology, Homerton Hospital, Homerton Row, London E9 6SR, UK.
Received 3 April 1990; and in revised form 10 July 1990.

These studies suggest that combined administration of uracil with Futraful might not only enhance antitumour activity, but also decrease systemic side effects. The results of a phase II trial of continuous daily administration of an oral preparation of Futraful and uracil (molar ratio 1:4), known as UFT, in patients with advanced colorectal and gastric carcinoma are reported here.

### Methods

### *Drug administration*

Phase I trials of UFT have established that the maximum tolerated dose is in the range of 12 mg $kg^{-1}$ $day^{-1}$ (Taguchi *et al.*, 1990). In this trial the dose given to patients was calculated on this basis, and given as three divided doses daily. In practice most patients received 600 mg UFT $day^{-1}$ (200 mg three times a day). Treatment was continued for at least three months, unless there was unequivocal disease progression or toxicity prior to formal radiological assessment of disease.

### *Patients*

The inclusion criteria for patients were as follows. (1) Pathologically confirmed diagnosis of metastatic colorectal or gastric cancer. (2) No prior chemotherapy in the three month period preceeding entry into the study. (3) No radiotherapy to sites of evaluable disease. (4) Evaluable disease in two dimensions as determined radiologically (CT Scan, Ultrasound). (5) Karnofsky performance status of 60 or greater. (6) Normal renal and hepatic function tests (unless due to disease), and adequate haemopoetic function (white cell count $> 4 \times 10^9 l^{-1}$, platelet count $> 100 \times 10^9 l^{-1}$).

### *Evaluation*

All patients were examined clinically prior to entry into the study. Baseline investigations included a full blood count, estimation of serum electrolytes, urea, creatinine, calcium, phosphate, bilirubin, alkaline phosphatase, SGOT, total proteins and albumin, and carcinoembryonic antigen. Evaluable disease was established by computerised axial tomography, or other appropriate investigations, e.g. ultrasound and chest radiographs.

Patients were evaluated clinically at 2 week intervals for the first month after commencing treatment, and monthly thereafter, with routine blood tests at each visit. Unless there was unequivocal disease progression or dropout due to drug toxicity, radiological re-evaluation of disease status was conducted at 3 monthly intervals. Standard WHO response criteria were used to assess response to treatment (WHO, 1979).

The details of the patients entered into the study are shown in Table I.

**Table I** Patient details

| | Colorectal cancer | Gastric cancer |
|---|---|---|
| Total number of patients | 40 | 18 |
| Evaluable | 36 | 16 |
| Sex | | |
| Male | 27 | 14 |
| Female | 13 | 4 |
| Age (years) | | |
| Mean | 59 | 63 |
| Range | (32–79) | (28–78) |
| Karnofsky performance status | | |
| Mean | 85 | 80 |
| Range | (70–100) | (70–100) |
| Sites of disease | | |
| Liver | 30 | 11 |
| Abdo/pelvis | 12 | 13 |
| Lung | 6 | 0 |
| Other | 3 | 1 |
| Previous chemotherapy | 3 | 1 |
| Previous radiotherapy | 3 | 0 |

### Results

### *Treatment duration*

The median duration of treatment in the colorectal cancer patients was 10 weeks (range 1–132 weeks), and 6 weeks (range 2.5–40 weeks) in patients with stomach cancer. Treatment discontinuation in all but two patients was due to progressive disease. These two patients who came off treatment due to toxicity were in the colorectal cancer group, and stopped treatment at 4 weeks (skin rash), and 32 weeks (peripheral neuropathy, hand-foot syndrome) respectively. A total of four (one toxicity, three progressive disease) patients in the colorectal cancer cohort, and two patients (progressive disease) in the gastric cancer cohort were unassessable because they completed less than four weeks of treatment.

### *Response to treatment*

No complete responses were seen in either the colorectal cancer or gastric cancer groups. There were six partial responses (16.6%; 95% confidence limits 6.4–32.8%) in the colorectal cancer group (Table II). The duration of response shown is from the time of formal assessment at 12 weeks and may therefore underestimate the actual duration of responses. All but one patient relapsed at the original metastatic site. Patient 2 had a sustained partial response in lung and liver metastases, but relapsed with histologically documented disease in the inguinal, supraclavicular, and mediastinal nodes. Patient 4 had to come off treatment due to toxicity (hand-foot syndrome and peripheral neuropathy). The median survival in the colorectal cancer patients was 34 weeks. No responses were seen in the previously treated patients.

There was only one partial response in the gastric cancer group, given a partial response of 6% (95% confidence limits 0.27–30.2%). This previously untreated patient had inoperable stomach cancer, because of disease extension behind the stomach. After 16 weeks of treatment with UFT, marked reduction of the abdominal disease was documented radiologically, and the patient was able to undergo a total gastrectomy. Pathological examination of the stomach revealed extensive fibrosis, with viable tumour present. The patient continued UFT, and remained well until 7 months after gastrectomy, when he was admitted with septicaemia and died. There was no evidence clinically of tumour, and his haematological indices did not suggest myelosuppression due to UFT. A post mortem was not held. The median survival in the gastric cancer group was 12 weeks.

**Table II** Details of patients with colorectal cancer responding to UFT

| Patient | Site of disease | Response duration | Site of relapse |
|---|---|---|---|
| 1 | Liver | 24 weeks | Liver |
| 2 | Lung and liver | 40 weeks | Lymph nodes |
| 3 | Liver | 3 weeks | Liver |
| 4 | Lung and liver | $>$20 weeks | Off treatment due to toxicity at 32 weeks |
| 5 | Liver | 24 weeks | Liver |
| 6 | Liver | 9 weeks | Liver |

### *Toxicity*

*Gastrointestinal toxicity* Mild nausea and vomiting was experienced by five patients at the start of treatment, but resolved with continuation of treatment. One patient had nausea and vomiting requiring treatment discontinuation temporarily. Two patients experienced diarrhoea, and in one patient this was severe enough to require cessation of treatment temporarily. UFT was restarted at 400 mg $day^{-1}$, and then increased to 600 mg $day^{-1}$ in this patient, and diarrhoea did not recur. Stomatitis was not seen in any patient.

*Skin* Skin toxicity was seen in six patients (16.6%). All of these patients developed itchy maculopapular eruptions on the trunk and forearms. In one patient the rash developed at 4 weeks and required treatment discontinuation. Treatment was not restarted in this patient because of disease progression. In the other patients, skin rashes developed after more than 12 weeks of treatment, and in two patients have persisted, although with improvement, despite treatment discontinuation for over 3 months. One of these patients has also developed marked skin pigmentation. One patient developed mild alopecia.

*Neurotoxicity* Two patients (5%) developed symptoms and signs of peripheral neuropathy at 32 and 36 weeks after commencing UFT. One of these patients also had signs of cerebellar dysfunction, which abated after cessation of treatment. Both these patients concomitantly developed the hand-foot syndrome. In both patients, this has abated following treatment discontinuation.

*Haematological toxicity* None of the patients had any evidence of treatment related anaemia or myelosuppression during treatment lasting up to 132 weeks. Of the 20 patients continuing therapy for 12 weeks or more, nine (45%) developed macrocytosis with normal serum and red cell folate, and serum vitamin B12 levels. Twenty-one patients (58%) had mild falls in their platelet counts at 4 weeks after starting therapy with none of these developing a platelet count less than $120 \times 10^9 l^{-1}$. One patient developed a platelet count of $40 \times 10^9 l^{-1}$ at 132 weeks of treatment, and treatment was discontinued. A bone marrow examination revealed mild decrease in cellularity. The platelet count in this patient continues to be low ($60 \times 10^9 l^{-1}$), 6 months after stopping UFT.

*Renal and hepatic toxicity* None of the patients in the trial had any evidence of treatment related liver or renal toxicity.

### Discussion

The overall prognosis for patients with advanced colorectal and gastric cancer remains poor, despite improvements in the response rates achieved with fluoropyrimidines by modifying administration schedules, and combining treatment with agents such as folinic acid and alpha-interferon (Wadler *et al.*, 1989). These regimes, although leading to complete responses in a small number of patients, are associated with appreciable toxicity, and require parenteral administration.

The results presented in this paper suggest that oral UFT is a relatively non-toxic chemotherapeutic agent that has some efficacy in the treatment of metastatic colorectal cancer, but a dissapointingly low rate of response in gastric cancer.

The observed response rate of 16.6% (95% confidence limits 6.4–32.8%), accurately documented with CT scanning of measurable disease, is within the range reported for standard bolus regimes of 5-FU, but lower than that observed with infusional 5-FU (Lokich *et al.*, 1989), or the combination of 5-FU with high dose folinic acid (Erlichman *et al.*, 1988). Although these regimes have led to complete remissions in a very small number of patients, they are associated with greater toxicity than UFT and for the majority of patients have no impact on survival. There is therefore still a need for a chemotherapeutic agent, which can be given with palliative intent, with a significant antitumour effect, ease of administration, and relatively few side effects. This study suggests that oral administration of UFT may fulfil some of the above criteria.

**References**

ANSFIELD, F., KLOTZ, J., NEALON, T. & 6 others (1977). A phase III study comparing the clinical utility of four regimens of 5-fluorouracil. *Cancer*, **39**, 34.

ANTTILA, M.I., SLOTANIEMI, E.A., KAIRALUOMA, M.I., MOKKA, R.E. & SUNDQUIST, H.T. (1983). Pharmacokinetics of Ftorafur after intravenous and oral administration. *Cancer Chemother. Pharmacol.*, **10**, 150.

AU, J.L., WU, A.F., FRIEDMAN, M.A. & SADEED, W. (1979). Pharmacokinetics and metabolism of Ftorafur in man. *Cancer Treatment Rep.*, **63**, 343.

BLOKHINA, N.G., VOSNY, E.K. & GARCIN, A.M. (1972). Results of treatments of malignant tumours with Ftorafur. *Cancer*, **30**, 390.

CHABNER, B.A. (1982). Pyrimidine antagonists In *Pharmacological Principles of Cancer Treatment*, Chabner, B.A. (ed.) p. 183. Saunders: Philadelphia.

DREWINKO, B. & YANG, L.Y. (1985). Cellular basis for the inefficacy of 5-FU in human colon carcinoma. *Cancer Treat. Rep.*, **69**, 1391.

EARL, H.M., COOMBES, R.C. & SCHEIN, D.S. (1984). Cytotoxic chemotherapy for cancer of the stomach. *Clin. Oncol.*, **3**, 351.

ERLICHMAN, C., FINE, S., WONG, A. & ELHAKIM, T. (1988). A randomised trial of fluorouracil with folinic acid in patients with metastatic colorectal carcinoma. *J. Clin. Oncol.*, **6**, 469.

FUJII, S., IKENAKA, K., FUKUSHIMA, M. & SHIRASAKA, T. (1978). Effect of uracil and its derivatives on antitumour activity of 5-fluorouracil and 1-(2-tetrahydrofuryl)-5-fluorouracil. *Gann*, **69**, 763.

FUJII, S., KITANO, S., IKENAKA, K. & SHIRASAKA, T. (1979). Effect of co-administration of uracil or cytosine on the antitumour activity of clinical doses of 1-(2-tetrahydrofuryl)-5-fluorouracil and level of 5-fluorouracil in rodents. *Gann*, **70**, 209.

FUJII, S., SHIRASAKA, T., IKENAKA, K. & KITANO, S. (1980). Effect of uracil on antitumour activity of 1-(2-tetrahydrofuryl)-5-fluorouracil. Current Chemotherapy and Infectious Disease: Proceedings of the 11th ICC and the 19th ICAAC, p. 1529.

LOKICH, J.J., AHLGREN, J.D., GULLO, J.J., PHILLIPS, J.A. & FRYER, J.G. (1989). A prospective randomised trial of continuous infusion fluorouracil with a conventional bolus schedule in metastatic colorectal carcinoma: a Mid-Atlantic Oncology Program Study. *J. Clin. Oncol.*, **7**, 425.

MOERTEL, C.G. (1973). Large bowel. In *Cancer Medicine*, Holland, J.F. & Frei, E. III (eds) p. 1597. Lea and Febiger: Philadelphia.

TAGUCHI, T., FURUE, H., KOYAMA, Y. & 4 others (1990). Phase I study of UFT. *Cancer Chemother*. (in the press).

TAGUCHI, T., NAKANO, Y., JIKUYA, K. & 4 others (1978). Determination of 5-fluorouracil levels in tumours, blood and other tissues. *Cancer Chemother.*, **5**, 1167.

WADLER, S., SCHWARTZ, E.L., GOLDMAN, M. & 6 others (1989). Fluorouracil and recombinant alpha-2a-interferon: an active regimen against advanced colorectal carcinoma. *J. Clin. Oncol.*, **7**, 1769.

WHO (1979). *Handbook for Reporting Results of Cancer Treatment*. Offset Publication no. 48, p. 22. WHO: Geneva.